# Gaseous NH_3_ Confers Porous Pt Nanodendrites Assisted by Halides

**DOI:** 10.1038/srep26196

**Published:** 2016-05-17

**Authors:** Shuanglong Lu, Kamel Eid, Weifeng Li, Xueqin Cao, Yue Pan, Jun Guo, Liang Wang, Hongjing Wang, Hongwei Gu

**Affiliations:** 1 College of Chemistry, Chemical Engineering and Materials Science, Collaborative Innovation Center of Suzhou Nano Science and Technology, Soochow University, Suzhou 215123, P. R. China; 2 State Key Laboratory of Electroanalytical Chemistry, Changchun Institute of Applied Chemistry, Chinese Academy of Sciences, Jilin 130022, P. R. China, University of Chinese Academy of Sciences, Beijing 100039, P. R. China; 3 School for Radiological and Interdisciplinary Sciences (RADX) & Collaborative Innovation Center of Radiological Medicine of Jiangsu Higher Education Institutions, Soochow University, Suzhou 215123, P. R. China; 4 Analysis and Testing Center, Soochow University, Suzhou 215123, P. R. China; 5 College of Chemical Engineering, Zhejiang University of Technology, Hangzhou 310014, P. R. China

## Abstract

Tailoring the morphology of Pt nanocrystals (NCs) is of great concern for their enhancement in catalytic activity and durability. In this article, a novel synthetic strategy is developed to selectively prepare porous dendritic Pt NCs with different structures for oxygen reduction reaction (ORR) assisted by NH_3_ gas and halides (F^−^, Cl^−^, Br^−^). The NH_3_ gas plays critical roles on tuning the morphology. Previously, H_2_ and CO gas are reported to assist the shape control of metallic nanocrystals. This is the first demonstration that NH_3_ gas assists the Pt anisotropic growth. The halides also play important role in the synthetic strategy to regulate the formation of Pt NCs. As-made porous dendritic Pt NCs, especially when NH_4_F is used as a regulating reagent, show superior catalytic activity for ORR compared with commercial Pt/C catalyst and other previously reported Pt-based NCs.

Morphology control of metal nanocrystals (NCs) is very critical to many important fields, such as fuel cells and fine chemical synthesis, because the unique activity of metal NCs can be easily tuned by tailoring their sizes and shapes^1^,^2^,^3^,^4^,^5^. It can be achieved through manipulating the reduction kinetics, heating, pH, ligands, or the addition of small amount of nucleating agents^6^,^7^,^8^. One of the most promising ways is to use gas atmosphere due to its ability to serve as a surface confining agent during the formation of NCs. For instance, freestanding Pd nanosheets were prepared with a CO-confined growth method and this extraordinary nanocrystals exhibited high performance in both electrocatalysis and photothermal therapy^9^. Lacroix *et al*. described the formation of Pt dendrites and fivefold stars under 3 bar H_2_ atmosphere^10^. They believed that H_2_ cannot only lower the reduction temperature but also “clean” the nanoparticle’s surface by temporarily removing weak coordinating ligands^11^. Therefore, the usage of gas environment is a promising strategy to achieve the morphology control.

Pt-based NCs are the most efficient catalysts for oxygen reduction reaction (ORR) in the proton-exchange membrane fuel cells (PEMFCs)^12^. However, the instability, high cost, and scarce of Pt metal are critical fences. Various efforts are dedicated to overcome these barriers including control Pt-based NCs morphology such as, nanospheres^13^, nanocubes^14^, nanowires^15^, mesoporous structures^16^, etc. Porous dendrites are among the most promising active and durable Pt NCs morphologies because they can provide sufficient active catalytic sites for reactant molecules. Also porosity facilitates molecular diffusion rate, leading to retard adsorption of poisoning species. Therefore, rational design and synthesis of porous Pt-based nanodendrites are of great significance to researchers. A lot of outstanding works have been done in the past few years. Seed growth method was the most studied pathway to facilitate the highly branched nanoparticles. Pd^17^, Au^18^, etc. can be used as premier nucleus. Recently, PtCu nanodendrites were obtained by templateless, and seedless hydrothermal method in the presence of poly (allylamine hydrochloride) and formaldehyde^19^. Route for the synthesis of dendritic Pt-Pd-Ru NCs were prepared by a one-step synthesis at room temperature without seed^20^. Formic acid and halides, as well, were both found to be qualified for regulating the formation of porous Pt nanodendrites^21^,^22^. In addition, methods like dealloying^23^,^24^,^25^, template synthesis^26^,^27^,^28^,^29^ and assembly strategies^30^ were often used to prepare Pt-based nanoporous structure. All of them are powerful and widely investigated strategies for generating Pt-based nanoporous structure^31^. Although the great progress in this area, developing facile methods to enable the controllable synthesis of porous Pt NCs with three-dimensional (3D) dendritic structure remains a challenge.

Herein, we report for the first time the synthesis of different 3D porous Pt nanodendrites under the NH_3_ gas atmosphere directing by different halides (shown in Fig. 1). Compared with other strategies, this novel method can afford highly uniform and nanoporous Pt dendrites efficiently. And also, different unique morphology of Pt can be obtained controllably.

NH_3_ gas is a frequently used ligand for Pt metal and it may function as a surface confining agent like CO and H_2_ gas, producing specific adsorption onto certain facet of Pt^32^. Also, ammonium halides NH_4_X (X = F, Cl, Br) were also used in regulating the formation of Pt NCs. Halide salts were previously reported by other groups in the synthesis of Au, Ag and Pd NCs^33^. The selective bonding of halide ions on specific facet of fcc metals has been demonstrated and it can be used to regulating the lateral growth rate. Moreover, the as-synthesized Pt NCs exhibit higher ORR activity and durability compared with commercial Pt/C catalyst.

## Results

### Synthesis and Characterization of Porous Pt Nanodendrites

The porous Pt nanodendrites were synthesized by controlled reduction of Pt acetylacetonate [Pt(acac)_2_] in oleylamine under NH_3_ gas atmosphere with adding different ammonium halides as regulating reagents, which was described in the supporting information. The porous nanodendrites obtained using F, Cl, Br as regulating reagents were denoted as NDs-F, NDs-Cl and NDs-Br respectively.

**Figure 2:**
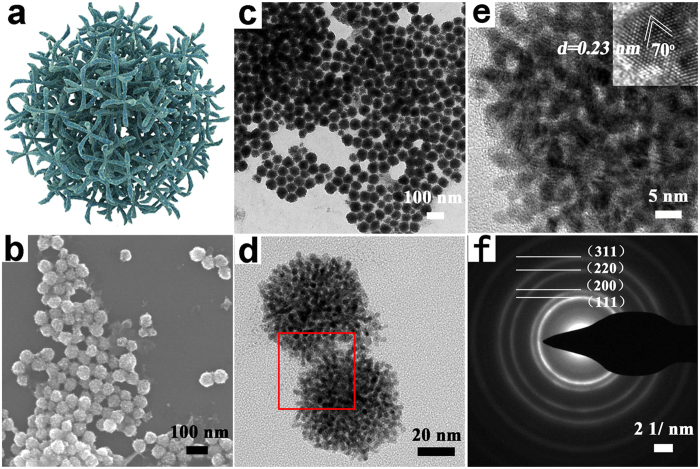
Structural characterizations of the porous Pt nanodendrites with NH_4_F as regulating reagent under NH_3_ atmosphere: (**a**) the model of the porous nanodendrite Pt NDs-F. (**b**) SEM image. (**c**) TEM image. (**d**) The higher magnification image of two individual porous nanodendrites. (**e**) High-resolution TEM image of an individual porous Pt nanodendrite taken from the region red-boxed in (**d**), the inserted is the higher magnification image showing the measured distance and angle of lattice plane. (**d**) SEAD pattern.

Figure 2b shows the typical scanning electron microscopy (SEM) image of well-dispersed porous nanodendrites when NH_4_F (2 mmol) was used as regulating reagent. The morphology of NDs-F is strikingly uniform and highly branched, which can also be confirmed by the transmission electron microscopy (TEM) image (Fig. 2c). These porous dendritic nanoparticles are assembled with numerous interconnected arms (2 ± 0.5 nm in width), which are spatially separated from each other. Notably, porous dendritic structures derived from these branched Pt arms are proved to be highly valuable for the enhancement of both catalytic activity and durability^13^,^14^,^15^,^16^,^17^,^18^,^34^. The high resolution TEM (HR-TEM) image shown in Fig. 2e further demonstrates the nanoporous structures nature of the NDs-F. As we can see, the lattice distance is 0.23 nm and the dihedral angle is around 70°, which is in good agreement with the (111) planes of Pt.

The X-ray diffraction (XRD) pattern profile of the as-synthesized nanomaterial shows a metallic face-centered (fcc) structure (Figure S1), composed of planes like (111), (200), (220) and (311). This is also consistent with the selected-area electron diffraction pattern (SAED) shown in Fig. 2f. And both of them indicate the well-crystallinity of the as-synthesized NDs-F. Also, Pt is the sole element that can be detected according to the SEM energy dispersive X-ray (SEM-EDX) profile shown in Figure S2.

### Roles of NH_3_ Gas in the Synthesis

NH_3_ gas is the key factor to the success of the synthesis. We firstly investigated the influence of NH_3_ gas by substituting NH_3_ with N_2_ in the preparation of NDs-F with other parameters remaining unchanged.

**Figure 3:**
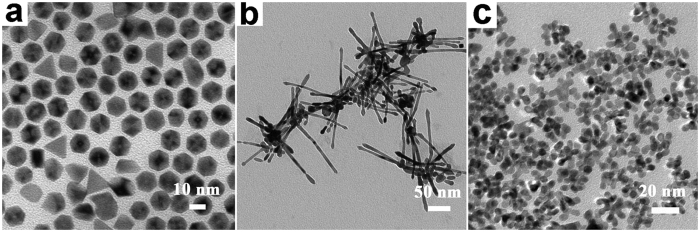
TEM images of samples two controlled trials: (**a**) 0.125 mmol Pt(acac)_2_, 2 mmol NH_4_F, 165 °C, 4 bar N_2_. (**b**) 0.125 mmol Pt(acac)_2_, 2 mmol NH_4_F, 165 °C, 1 bar NH_3_. (**c**) 0.125 mmol Pt(acac)_2_, 165 °C, 4 bar NH_3_.

As evidenced in Fig. 3a, only well-dispersed Pt nanoparticles (10 ± 2 nm) can be obtained when the NH_3_ gas was absent. Further control experiment was performed by lowering the NH_3_ pressure to normal atmosphere. As we can see, the highly porous and dendrite structures have almost vanished, which were been replaced by interconnected smooth Pt nanorods (100 ± 5 nm in length) shown in Fig. 3b. Moreover, when only NH_3_ gas was used without NH_4_^+^ salts added (Fig. 3c), we can obtained dendritic Pt nanoparticles.

Based on these observation, possible roles are raised here to illustrate the crucial status of ammonia gas. Both the oleylamine and NH_3_ gas have the ability to bond strongly onto the surface of Pt nanoparticles^35^, which, we suppose, will give rise to a competitive mechanism. When the NH_3_ gas was absent, only the solvent oleylamine had absorption on the surface (take no account of halides) and excellent performance of it in surface capping resulted in the well-defined nanoparticles without branches. After co-absorption of oleylamine and NH_3_ gas by applying NH_3_ gas flow into the reaction mixture, branches started to emerge because NH_3_ gas is strong enough to complete with oleylamine and NH_3_ molecular, who is free from long aliphatic chain, may favor the anisotropic growth. When the NH_3_ gas pressure was further increased to 4 atm, the as-synthesized Pt NCs were totally well-defined NDs-F. Combined with the truth we can obtain dendritic Pt nanoparticles when only NH_3_ gas was used, we can draw the conclusion that it is the NH_3_ gas, not NH_4_^+^ ion, is vital for the formation of porous Pt nanodendrites. Also, the NH_3_ pressure makes big differences here. Moreover, on the basis of previous research, the thermal reduction of Pt(acac)_2_ in pure oleylamine at lower than 180 °C could hardly took place^36^, suggesting throughout our synthesis, the presence of NH_3_ gas is favorable for the reduction of Pt precursor, which is another and rarely investigated role of the NH_3_ gas. This hypothesis was further evidenced that we found a sharp decrease of pressure once the autoclave charged with NH_3_ gas was placed into the oil bath, while the same phenomenon cannot be found when the autoclave was charged with N_2_ gas. Although the concrete reason remains unclear, possible coordination of NH_3_ with Pt^2+^ to form entirely new complex may be responsible for it. New complex lowers the reduction potential of Pt precursor and eventually lowers the reaction temperature. Generally, interactions of the NH_3_ gas with both Pt precursors and NC intermediates contribute to the possible coordination and competitive mechanisms respectively, which demonstrates the NH_3_ gas-directing strategy is indispensable in this novel synthesis of porous Pt nanodendrites.

### Roles of Halides in the Synthesis

It has also been widely accepted that halide ions played a critical role in making metal structures and however, it is not yet clear that which potential halide-metal interaction is decisively responsible for the shape control of nanostructures^18^,^19^,^20^,^37^,^38^,^39^. Recently, Murphy and his coworkers have summarized the role of halides in synthesis of anisotropic noble metal, such as absorbate, completing agent and surfactant micelle controller^40^. Herein, to see what will happen if we change the halide ion in our synthesis, we used Cl^−^ as the alternative directing reagent for comparison.

**Figure 4:**
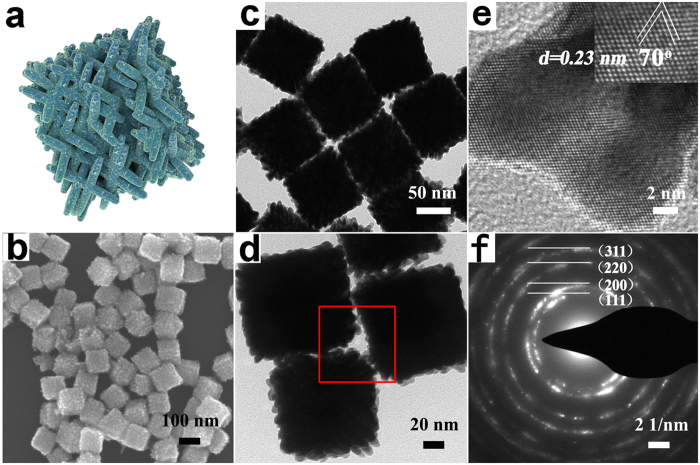
Structural characterizations of the porous Pt nanodendrites with NH_4_Cl as regulating reagent under NH_3_ atmosphere: (**a**) the model of the porous nanodendrite Pt NDs-Cl. (**b**) SEM image. (**c**) TEM image. (**d**) TEM image of three individual porous nanodendrites. (**e**) High-resolution TEM image of one single exposed branch of the porous nanodendrite taken from the region red-boxed in (**d**), the inserted is the higher magnification image showing the measured distance and angle of lattice plane. (**f**) SEAD pattern.

**Figure 5:**
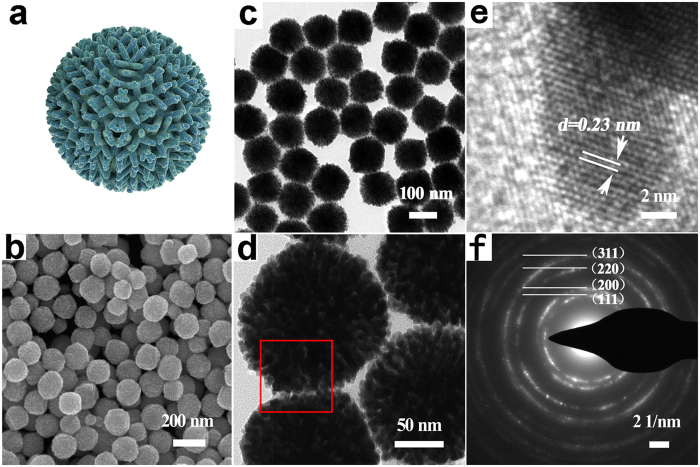
Structural characterizations of the porous Pt nanodendrites with NH_4_Br as regulating reagent under NH_3_ atmosphere: (**a**) the model of the porous nanodendrite Pt NDs-Br. (**b**) SEM image. (**c**) TEM image. (**d**) TEM image of individual porous nanodendrites. (**e**) High-resolution TEM image of one single exposed branch of the porous nanodendrite taken from the region red-boxed in (**d**), the inserted is the higher magnification image showing the measured distance. (**f**) SEAD pattern.

As indicated in Fig. 4, the as-synthesized NDs-Cl are cubic branched assemblies (120 ± 5 nm in size), which are rarely seen in previous reports. Compared with NDs-F, these 3D assemblies are twice larger in size, cubic porous in their shapes and much denser in their structures. Even though they still reserve the nanoporous patterns, their component interconnected arms become bolder than before, as a result of which, the size of the mesopores shrink correspondingly. Further investigation was conducted using Br^−^ as addictive (Fig. 5). The size of NDs-Br are further enhanced (140 ± 5 nm) and the arms become bolder than ever. Quite different from the cubic-like nanoporous NDs-Cl, the as-obtained nanodendrites are porous ball-like 3D assemblies.

Inspired by the dual-function of NH_3_ gas we mentioned before, we build our hypothesis for the roles of halides in our synthesis on a combination of interaction of halides with both Pt precursors and NC intermediates as well. For one thing, interaction between the halides and Pt precursors will slow the Pt nanoparticle growth. Lower reduction rate is favorable for inducing relatively big-sized and highly branched nanostructures^2^. In our synthesis strategy, the situations where Cl^−^, Br^−^ were used as shape-directing agents saw higher reduction potentials and lower reduction rates compared with where F^−^ was used. Consequently, the size of single nanoparticle was larger and the anisotropic shape was much more unique correspondingly. For another, interaction between the halides and Pt NC intermediates should be taken into consideration because halides have a preferential absorption onto certain low-index facets. The binding strength and tendency vary from one halide ion to another. Although it is different from each other that the stabilization of certain facet with breaking the fcc cubic symmetry to achieve anisotropic NPs, the selective bonding of halides still has a important role in local shape evolution^40^. It will serve as a completing reagent with NH_3_ gas and the oleylamine, changing the energetic sequence of those low index facets. Then, giant differences aroused from those subtle energy changes will be made to final morphologies of Pt.

### Halide Binding Behavior on Pt (111) Facet from Theoretical Calculation

To verify the preferential absorption behavior onto certain Pt low-index facets, density function theory (DFT) calculations were uesd to provide a in-depth view at atomic level. We have examined the binding process of F, Cl and Br on the representative Pt (111) facet. The Pt surface model contains 7 Pt atomic layers as illustrated in Figure S3A. The bottom three layers (pink colored balls) are fixed to mimic the bulk effect. Accounting the structural symmetric of Pt (111) surface, there are two representative binding sites for a foreign atom as demonstrated in Figure S3B: site 1 is just on the top of a surface Pt atom; site 2 is on the top of triangle formed by three surface Pt atoms. In order to quantify the binding strength, the binding energy is defined as


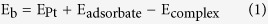


Where E_Pt_ is total energy of the bare Pt surface, E_adsorbate_ is the potential energy of the adsorbate atom of F, Cl, Br and Pt, E_complex_ is total energy of Pt surface with the adsorbate. According to this definition, a larger value of E_b_ denotes a more stable binding.

The DFT calculations reveal distinct binding behaviors among the three adsorbates as shown in Figure S3C. For F, it can only bind at site 1, that is, just on the top of a surface Pt atom. If F is placed on site 2 at the beginning, it will migrate to site 1 quickly during structural optimization. More importantly, F forms the strongest, mono-valent bond with a Pt, with a binding energy reaching 3.25 eV. For Cl, it still favors the site 1 with binding energies of 2.80 eV (on site 2, the value decreases to 2.55 eV). For the case of Br, the stable binding site has changed to be site 2. In detail, the binding energies are 2.30 (on site 1) and 2.44 eV (on site 2), respectively. The change of the favored binding site from F to Br should be attributed to the electronegativity of these elements. F is the most electronegative element which prefers to form a monovalent bond. As a consequence, F tends to locate on the top of a Pt atom. For Br, it behaves more like a “metal” by sharing its electrons with adjacent Pt neighbors. Hence a Br will occupy the site 2 (on the top of the three Pt triangles) which has three coordinations.

For comparison, a Pt atom binding at site 2 (representation the Pt cluster growing process) is selected as the control study. The bonding energy for such a process is 4.62 eV from our calculation. Considering that F’s binding energy reaches 3.25 eV, it is obvious that F is almost compatible to Pt’s binding. Hence the existence of F will inhibit Pt growth regardless of the specific direction, which can be treated as “isotropic” effect. This results in the “loose spherical” shape of the Pt cluster.

For the case of Cl and Br, the binding is relatively weaker than F. Thus Cl and Br are less capable to inhibit Pt growth. Overall, certain directions can still be well crystallized during growth and thus Pt cluster becomes more “regulate” with the existence of Cl or Br. However, as revealed from binding site calculations, Cl and Br have distinct binding sites on Pt surface. This will affect Pt growth only on specific directions, resulting in the different morphology of the Pt clusters for Cl and Br as observed in the experiment.

### Tracking Study of the Shape Evolution of Pt NDs

**Figure 6:**
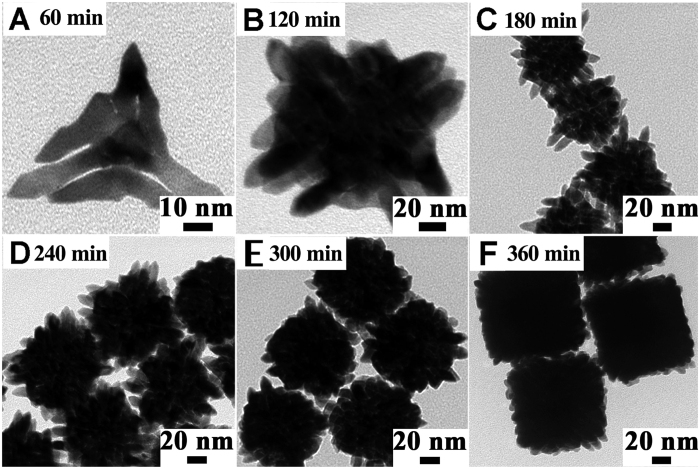
TEM images of samples taken from the quenched solutions at different stages of the synthesis process of Pt NDs-Cl (0.125 mmol Pt(acac)_2_, 2 mmol NH_4_Cl, 165 °C, 4 bar NH_3_) : (**A**) 60 min. (**B**) 120 min. (**C**) 180 min. (**D**) 240 min. (**E**) 300 min and (**F**) 360 min.

Upon the reveal of the roles of NH_3_ gas and halides, time-dependent growth process was also observed by TEM imaging at different stages of the formation of Pt NDs-Cl as an example (Fig. 6A–F) (the results for Pt NDs-F and Pt NDs-Br are shown in the supporting information).

As we can see, at the initial stage (after 1 h), several thick stems (4 ± 1 nm in width) extended from the seed core. Then, following spatial growth continued to proceed, producing more stems out and also more interconnected arms. Much more complex structures were obtained in the following 5 hours until the color of the reaction mixture remained stable deep black, which indicated the complete consumption of the precursor. Obviously, random aggregation of the single Pt branches can be ruled out in this situation, which can also be confirmed in Fig. 4, continuous lattice fringes are stretching from the core to branches.

Notably, an anisotropic autocatalytic growth, which has been reported in some previous researches^1^,^17^,^18^, may be involved. The experimental condition was not strong enough to completely reduce Pt ions to Pt atom. The interaction of NH_3_ molecules or halides with Pt atoms or ions will lead unreacted Pt ions to orient towards the surface of entirely evolved Pt atoms. And thereby, Pt ions can be reduced on the surface catalyzed by Pt atoms. This is consistent with our observation that the rate of the first stage (0–1 h, Fig. 6A) during the synthesis was quite slow. Afterwards, the reduction rate paced up obviously in the following hours (Fig. 6B–F), until the exhaustion of precursors.

### Electrocatalytic Applications

**Figure 7:**
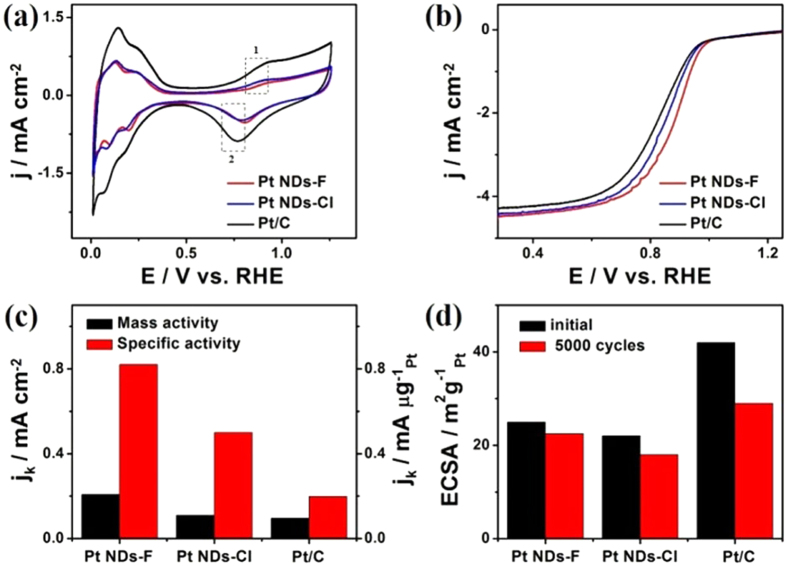
(**a**) CVs at a scan rate of 50 mV s^−1^ and (**b**) ORR polarization curves at a scan rate of 10 mV s^−1^ with a rotation rate of 1600 rpm of the three catalysts measured in a N_2_ saturated- and an O_2_ saturated- 0.1 M HClO_4_ solution, respectively. The dotted box 1 and 2 in (**a**) represent the potentials of OH_ad_ adsorption/desorption respectively. (**c**) Comparisons of the specific and mass activities at 0.9 V vs. RHE. (**d**) Comparisons of ECSAs.

The ORR activity of Pt NDs-F is investigated and benchmarked against Pt NDs-Cl and the commercial Pt/C catalyst. The CVs curves for the catalysts show distinctive potential regions H_upd_ /H_des_ (H_upd_ = H^+^ + e^−^) and OH_des_ (OH_ad_ + H_3_O^+^ + e^−^ = 2H_2_O) (Fig. 7a). The potentials of OH_ad_ adsorption/desorption peaks are (0.93/0.81 V) for Pt NDs-F, which positively shifted compared with those of Pt NDs-Cl (0.91/0.79 V) and Pt/C (0.92/0.77 V) (Fig. 7a). This is suggesting the delayed formation and weakening of Pt-oxygenated species onto porous nanodendrites relative to Pt/C, a desired feature for a good ORR catalyst in agreement with reports elsewhere^20^,^41^,^42^,^43^,^44^,^45^,^46^,^47^,^48^,^49^,^50^,^51^,^52^. The electrochemically active surface areas (ECSAs) of Pt NDs-F (25 m^2^ g^−1^_Pt_) and Pt NDs-Cl (22 m^2^ g^−1^_Pt_) are about 59 and 52% of the Pt/C (42 m^2^ g^−1^_Pt_). Notably, Pt NDs-F and Pt NDs-Cl provide high surface areas despite their relatively large overall particle size due to their porous and branched structure, which are not vulnerable to aggregation. This result is in consistence with previous reports related to either porous Pt or Pt-based catalysts^42^,^43^,^44^,^45^,^48^,^53^,^54^,^55^,^56^,^57^,^58^,^59^,^60^.

ORR polarization curves display that, the half-wave potentials of Pt NDs-F (0.87 V) is positively shifted relative to Pt NDs-Cl (0.83 V) and Pt/C (0.80 V) (Fig. 7b). The Pt NDs-F exhibit a mass activity of 0.207 mA μg^−1^_Pt_ which is 1.88 and 2.17 times higher than those of the Pt NDs-Cl (0.11 mA μg^−1^_Pt_) and Pt/C (0.095 mA μg^−1^_Pt_) at 0.9 V (Fig. 7c). The specific activity of Pt NDs-F (0.82 mA cm^−2^) is 1.64 folds of that of the Pt NDs-Cl (0.5 mA cm^−2^) and 4.14 folds of that of the Pt/C (0.198 mA cm^−2^). Pt NDs-Br exhibits insignificant ORR activity relative to other catalysts. Specifically the mass activity and specific activity of Pt NDs-Br are 0.062 mA μg^−1^_Pt_ and a 0.11 mA cm^−2^ respectively. It is intriguingly that, the mass activity of Pt NDs-F is superior to the reported mesostructured Pt (0.04 mA μg^−1^_Pt_)^40^ and porous Pt nanotubes (0.088 mA μg^−1^_Pt_)^61^, measured under similar conditions. The slightly shift in the diffusion limit currents in ORR polarization curves, most probably attributed to poor quality of the deposited films on the rotating glassy carbon disk electrode (RDE) in agreement with various previous reports^46^,^62^,^63^,^64^. Hence we performed multiple tests on many catalytic films for reliable comparison of the activity and durability for the different catalysts (Table S1).

The instability of Pt-based catalysts is a critical issue inhibits the widespread application of PEMFCs, due to spontaneous dynamic altering of Pt NCs surface via oxidation/reduction during the operation cycles^60^. The ECSAs of three catalysts were measured initially and immediately following 5000 accelerated durability test cycles. Interestingly, Pt NDs-F, Pt NDs-Cl and Pt/C reserve around 90%, 81% and 69% of their initial ECSA after 5000 cycles, respectively (Fig. 7d). Furthermore, Pt NDs-F, Pt NDs-Cl and Pt/C display 3, 8 and 18 mV degradation, respectively, in the half-wave potentials after 5000 cycles (Figure S6b,d,f).

Overall, Pt NDs-F, Pt NDs-Cl exhibited superior catalytic activity and durability relative to those of Pt/C. This is ascribed to dissimilarity in the morphology, composition, and surface facets among three catalysts. Particularly, Pt NDs-F, Pt NDs-Cl provides high ECSA owing to their porous morphology. Also, multi-rough branches of porous Pt NDs-F, Pt NDs-Cl afford massive adsorption catalytic sites for O_2_ molecules that are beneficial toward ORR. This led to protection the active catalytic sites from blocking by the poisoning species. Additionally, 3D porous nanodendritic morphology involved various 1D branches assembled together is less susceptible to dissolution, Ostwald ripening, and aggregation relative to 0D Pt on Pt/C. Although carbon supporter enhance the ECSA of Pt/C, it suffers from inevitable corrosion, which causes detachment of Pt nanoparticles, Ostwald ripening, and aggregation compared with supportless Pt catalyst. This subsequently led to massive loss of the ECSA and ORR activities. Meanwhile, our obtained porous nanodendritic Pt catalysts compose of massive assembled Pt nanocrystals with the same crystal orientation enclosed with high index facets {111}, {311}, {200} and {220}. This retard OH_ads_ species relative to low-index planes of Pt nanoparticles on Pt/C that is enclosed with {111} planes (Figure S7)^50^. Therefore, our newly designed porous nanodendrites Pt catalyst with their novel porous morphologies can eliminate the need for carbon supported Pt catalysts.

## Discussion

In conclusion, we have developed an efficient strategy to selectively synthesize uniform porous Pt nanodendrites with various morphologies co-assisted by NH_3_ gas and halides. By adjusting the halides (F^−^, Cl^−^ and Br^−^) in the synthesis, the morphologies of the porous Pt NCs are tuned. It is the first time the NH_3_ gas is used to assist the formation of Pt NCs. Also, a combination of interaction of NH_3_ molecules or halide ions with both Pt precursors and NC intermediates is proposed here to illustrate the roles of NH_3_ gas and halides we used. The as-synthesized porous Pt nanodendrites, especially when NH_4_F is used as a regulating reagent, show superior catalytic activity and durability for ORR compared with commercial Pt/C catalyst and other previously reported Pt-based NCs.

## Methods

### Chemicals and Materials

Oleylamine (OAm, >70%) and hexane (99.5%), were both purchased from Tokyo Chemical Industry Co. Ltd., Shanghai. Pt(acac)_2_ (99.99%), Nafion (5% w/w solution) were all obtained from Alfa Aesar. Ammonium fluoride (≥96%), Ammonium chloride (≥99.5%), Ammonium bromide (≥99%), chloroform (≥99%) and ethanol (≥99.5%) were all purchased from Sinopharm Chemical Reagent Co. Ltd. All the chemicals were of analytical grade and used as received without further purification.

### Synthesis of Porous Pt Nanodendrites

In a typical synthetic process, NH_4_X (X = F, Cl, Br) (2 mmol) was added into oleyamine (31 mmol, 10 ml) in a three-necked flask, followed by stirring for 30 min at 100 °C in N_2_ atmosphere to make sure the NH_4_X dissolved thoroughly. Pt(acac)_2_ (0.125 mmol, 49.2 mg) was then added to the mixture and another 30 min was taken to make it uniform. The solution was cooled to room temperature and was transferred into an autoclave, which was then charged with NH_3_ to 4 bar. The autoclave was heated to 165 °C and kept at this temperature for 4 h with stirring. The resulting black colloidal products was collected by centrifugation and washed several times with ethanol and chloroform. The final Pt crystals were dispersed in hexane for further use.

### Materials Characterization

The morphology of porous Pt nanodendrites were obtained by a transmission electron microscopy (TEM) (TecnaiG220, FEI, American) equipped with a Gatan CCD794 camera operated at 200 KV. High-resolution TEM (HRTEM), Energy dispersive X-ray (EDX) of a single nanohybrid (TEM-EDX) and SAED were carried out on a Tecani G2 F20 instrument at an accelerating voltage of 200 KV. SEM and SEM-EDX spectroscopy were performed on a Hitachi S-4700 cold field emission scanning electron microscope operated at 30 KV. The powder wide angle X-ray diffraction pattern (XRD) were recorded on an X’Pert-Pro MPD diffractometer (Netherlands PANalytical) with a Cu Kα X-ray source (λ = 1.540598 Å). The Pt loading amount in every catalysts was determined by using the inductively coupled plasma optical emission spectrometry (ICP-OES) analysis was conducted using a Thermo Scientific iCAP6300 (Thermo Fisher Scientific, US).

### Density Functional Theories Calculation

All the calculations were perfored using Vienna *ab initio* simulation package (VASP)^65^,^66^. Projector-augmented-wave (PAW) potentials were used to take into account the electron-ion interactions, while the electron exchange-correlation interactions were treated using a generalized gradient approximation (GGA) in the scheme of Perdew-Burke-Ernzerhof^67^,^68^. A plane wave cut off of 500 eV was used for all the calculations. Atomic relaxation was performed until the change of total energy was less than 0.01 meV and all the forces on each atom were smaller than 0.01 eV/Å, which was sufficient to obtain relaxed structures. A *k*- point sampling of 5 × 5 × 1 was used for the structure relaxation, while a denser mesh of 11 × 11 × 1 was used to calculate energies.

### Electrochemical Investigations

Cyclic voltammograms (CVs) and linear sweep voltammograms (LSVs) experiments were performed using a CHI 832 C electrochemical analyzer (Chenhua Co., Shanghai, China). A conventional three-electrode cell was used, including an Ag/AgCl (saturated KCl) electrode as reference electrode, a Pt wire as counter electrode and a working electrode. The working electrode was a modified rotating glassy carbon disk electrode (RDE). The modified RDE was coated with different catalysts with the same loading of 15 μg and dried at room temperature, and then, 3 μL of Nafion (0.05%) was coated on the surface and dried before electrochemical experiments. ORR measurements were performed on a RRDE-3 A rotation system (ALS Co. Ltd., Japan) with modified RDE in an O_2_-staurtaed aqueous solution with 0.1 M HClO_4_ at a rotation rate of 1,600 rpm with a scan rate of 10 mV s^−1^. ORR durability tests were conducted with a rotation rate of 1,600 rpm at a scan rate of 10 mV s^−1^ for 10,000 cycles. All the potentials are reported with respect to the reversible hydrogen electrode (RHE). Current densities were normalized by the working electrode geometric area. Specific activity and mass activity were respectively normalized by the ECSAs and the loading amount of Pt,

The ECSA can be calculated by the following equation: ECSA = *Q*_H_/m × 210, where, *Q*_H_ is the charge for H_upd_ adsorption determined using *Q*_H_ = 0.5 × *Q*, where *Q* is the is the charge in the H_upd_ adsorption /desorption area obtained after the double layer correction region, between 0 and 0.37 V, m is the Pt loading amount on the electrode, and 210 μC cm^−2^ is the charge required for monolayer adsorption of hydrogen on Pt surface.

The Koutecky-Levich equation was used to calculate the kinetic current, which can be described as follow:


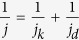


Where *j* is the measured current density, *j*_*k*_ and *j*_*d*_ are the kinetic and diffusion-limited current densities, and then, the kinetic current was calculated based on the following equation:


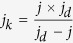


## Additional Information

**How to cite this article**: Lu, S. *et al*. Gaseous NH_3_ Confers Porous Pt Nanodendrites Assisted by Halides. *Sci. Rep*. **6**, 26196; doi: 10.1038/srep26196 (2016).

## Supplementary Material

Supplementary Information

## Figures and Tables

**Figure 1:**
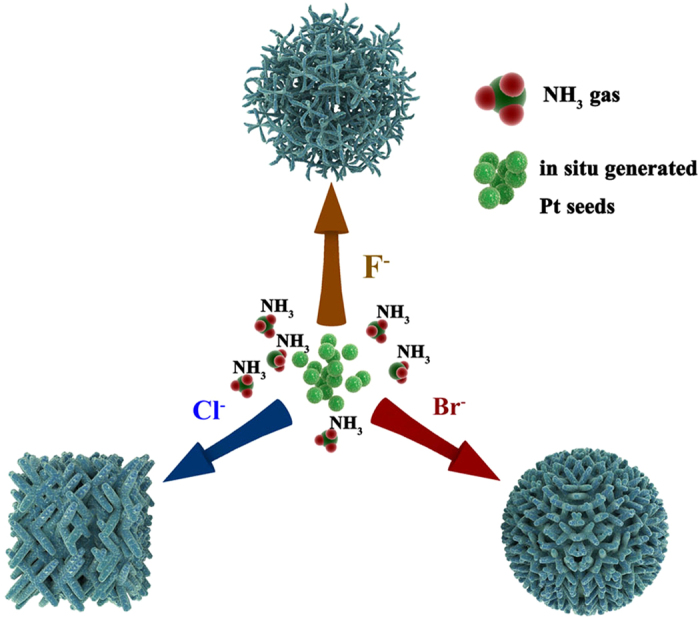
Illustration the synthesis of different porous nanodendrites under ammonia gas directed by different halide ions respectively.
