# Supplementary material for: Gaseous NH3 Confers Porous Pt Nanodendrites Assisted by Halides
Source: Sci Rep. 2016 May 17;6:26196. doi: 10.1038/srep26196 (PMC4869020; doi:10.1038/srep26196)
Supplement: Supplementary Information [file srep26196-s1.doc]

**Gaseous NH_3_ Confers Porous Pt Nanodendrites Assisted by Halides**

**Shuanglong Lu^a^, Kamel Eid^b^, Weifeng Li^c^, Xueqin Cao^a^, Yue Pan^a^, Jun Guo^d^, Liang Wang^e^, Hongjing Wang, ^e^* Hongwei Gu^a^***

*^a^ College of Chemistry, Chemical Engineering and Materials Science, Collaborative Innovation Center of Suzhou Nano Science and Technology, Soochow University, Suzhou 215123, P. R. China*

*E-mail:* [*hongwei@suda.edu.cn*](mailto:hongwei@suda.edu.cn)

*^b^ State Key Laboratory of Electroanalytical Chemistry, Changchun Institute of Applied Chemistry, Chinese Academy of Sciences, Jilin 130022, P. R. China*

*University of Chinese Academy of Sciences, Beijing 100039, P. R. China*

*^c^ School for Radiological and Interdisciplinary Sciences (RADX) & Collaborative Innovation Center of Radiological Medicine of Jiangsu Higher Education Institutions, Soochow University, Suzhou 215123, P. R. China*

*^d^ Analysis and Testing Center, Soochow University, Suzhou 215123, P. R. China*

*^e^ College of Chemical Engineering, Zhejiang University of Technology, Hangzhou 310014, P. R. China*

*E-mail:* [*hjw@zjut.edu.cn*](mailto:hjw@zjut.edu.cn)

**Modeling for the DFT calculation**

First, the fcc Pt lattice were calculated to be 2.821Å, which is good consistant with the experimental value. Hereafter, the primitive cell was replicated to generate the (111) surface model as illustrated in Figure S3. The model contains 112 Pt atoms. In the z direction, a vacuum space of 20 Å was kept to mimic the Pt surface. All the three dimensions and the 3-bottom Pt atomic layers (red-colored atoms in Figure S3A) are fixed to bulk value during structural optimization.

**Figure S1.** XRD profiles for NDs-F, NDs-Cl and NDs-Br respectively.


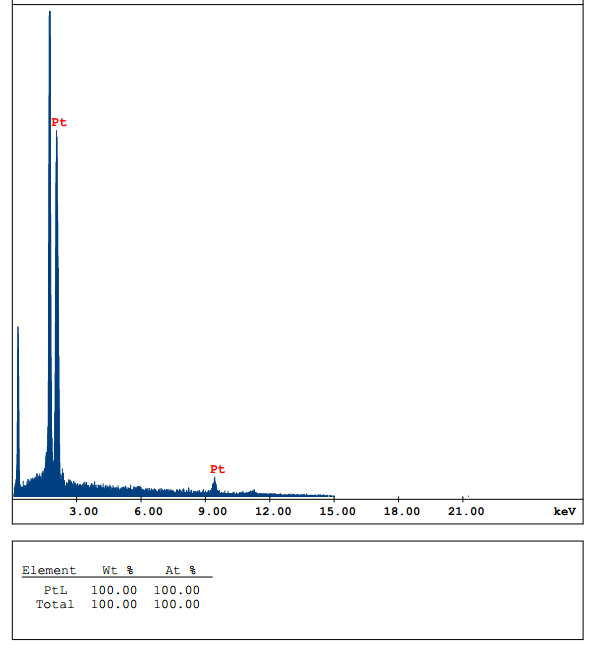


**Figure S2.** SEM-EDX profiles of NDs-F.


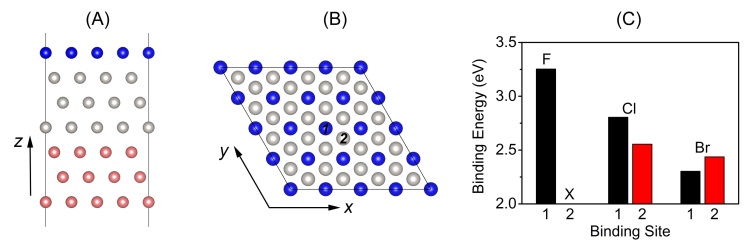


**Figure S3.** (A) side view and (B) top view of Pt (111) surface; the blue-colored Pt indicate surface atoms, and pink-colored Pt atoms are frozen during structural optimization to mimic the bulk effect; accounting the structure symmetric, there are two representative binding sites 1 and 2;(C) binding energies of adsorbates F, Cl and Br on Pt surface on two binding sites,. For the reference, a Pt atom binding at the site 2 has a binding energy of 4.62 eV.


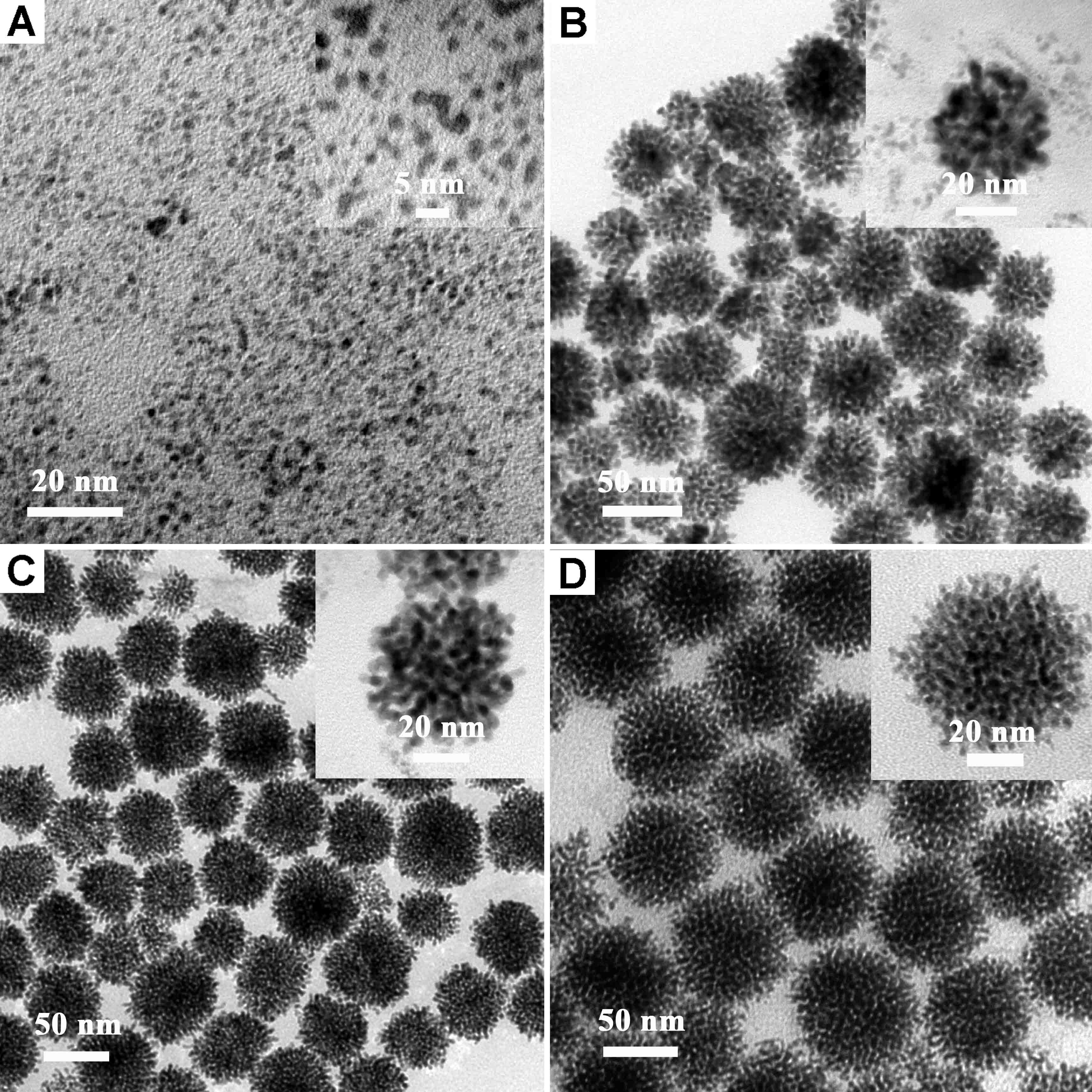


**Figure S4.** TEM images of samples taken from the quenched solutions at different stages of the synthesis process of Pt NDs-F (0.125 mmol Pt(acac)_2_, 2 mmol NH_4_F, 165 °C, 4 bar NH_3_) : (A) 60 min. (B) 120 min. (C) 180 min. and (F) 360 min.


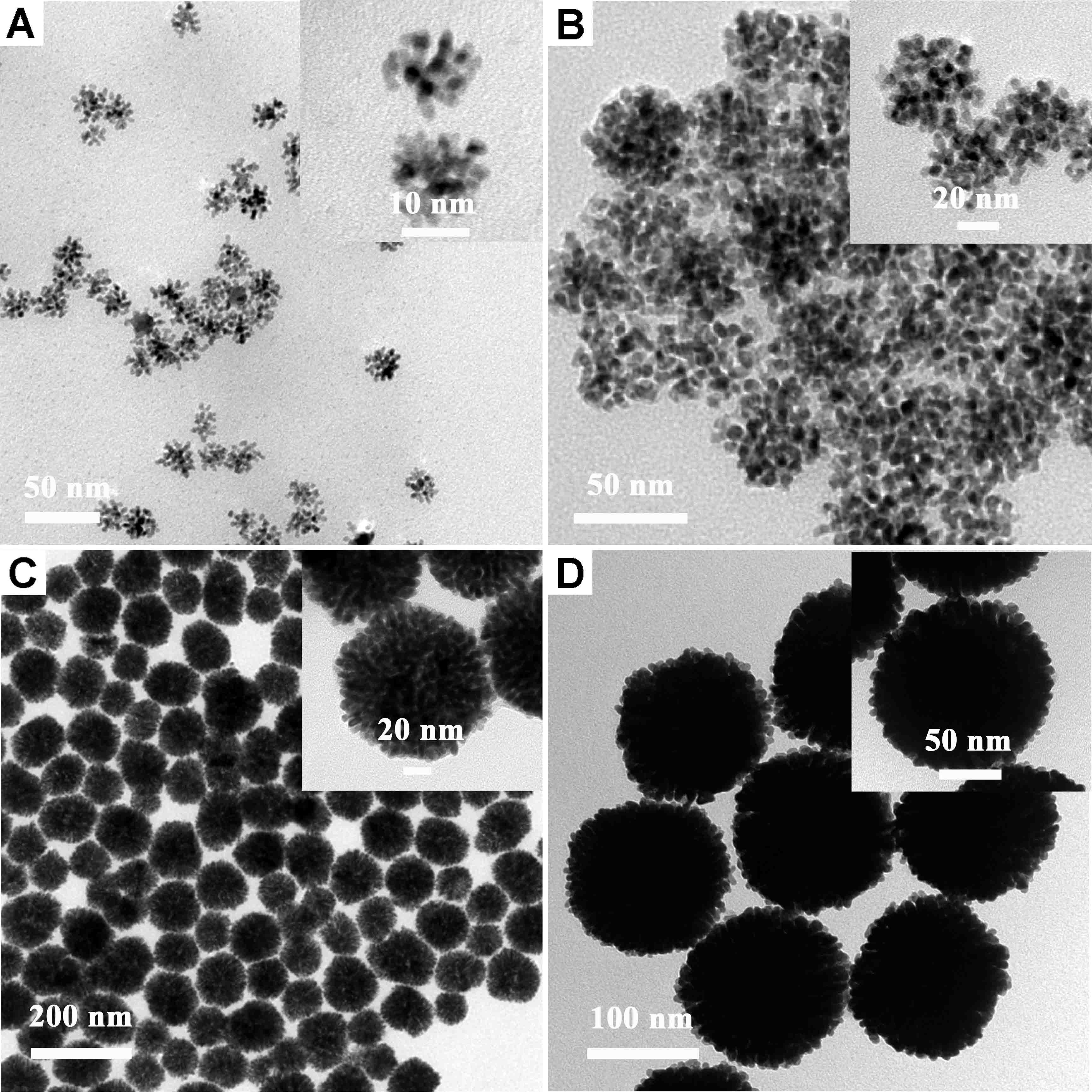


**Figure S5.** TEM images of samples taken from the quenched solutions at different stages of the synthesis process of Pt NDs-Br (0.125 mmol Pt(acac)_2_, 2 mmol NH_4_Br, 165 °C, 4 bar NH_3_) : (A) 60 min. (B) 120 min. (C) 180 min. and (F) 360 min.

**Table S1.** Comparison of ECSA and ORR activities measured at 0.9 V versus RHE for Pt NDs-F, Pt NDs-Cl, and l Pt/C catalysts.

| Catalyst | loading (µg cm^-2^) | ECSA (m^2^ g^-1^_Pt_) | Mass activity (mA µg^-1^_Pt_) | Specific activity (mA cm^-2^) |
| --- | --- | --- | --- | --- |
| Pt NDs-F | 15 | 25 ± 4.1 | 0.207 ± 0.043 | 0.82 ± 0.1 |
| Pt NDs-Cl | 15 | 22 ± 3.3 | 0.11 ± 0.025 | 0.5 ± 0.08 |
| Pt/C | 15 | 42 ± 6.2 | 0.095 ± 0.033 | 0.198 ± 0.05 |


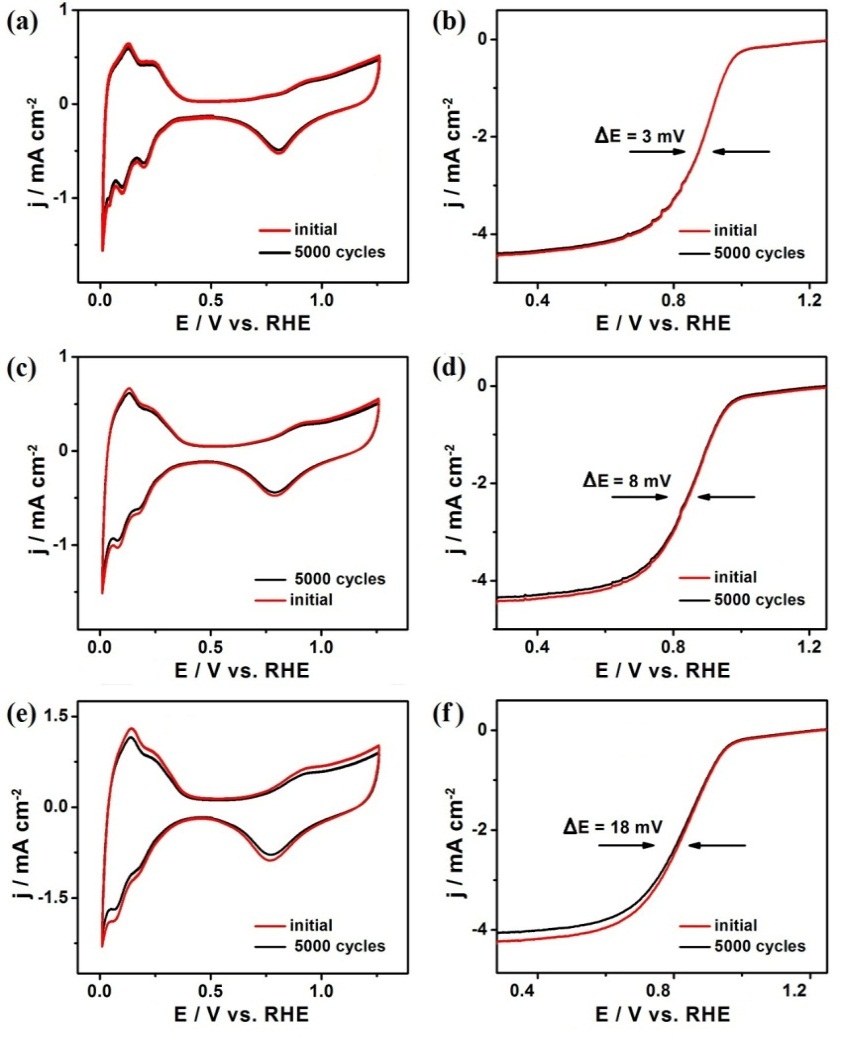


**Figure S6.** (a, c and e) CVs at a scan rate of 50 mV s^-1^ measured in a N_2_ saturated 0.1 M HClO_4_ solution and (b, d and f) ORR polarization curves before and after durability tests measured in an O_2_ saturated 0.1 M HClO_4_ solution at a scan rate of 10 mV s^-1^ with a rotation rate of 1600 rpm at a potential of 0.9 V for the Pt NDs-F (a and b), Pt NDs-Cl (c and d) and Pt/C (e and f).


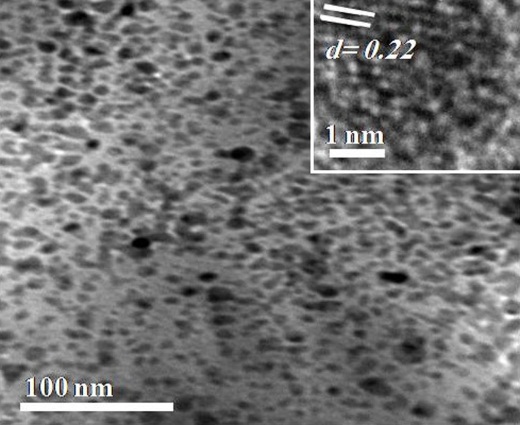


**Figure S7.** TEM image of Pt/C and its HR-TEM shown in the inset.
